# 1,4-Bis[(2,2′:6′,2′′-terpyridin-4′-yl)­oxy]butane

**DOI:** 10.1107/S1600536811050343

**Published:** 2011-11-30

**Authors:** Matthew P. Akerman, Craig D. Grimmer, Varvara I. Nikolayenko, Desigan Reddy

**Affiliations:** a School of Chemistry, University of KwaZulu-Natal, Private Bag X01, Pietermaritzburg 3209, South Africa

## Abstract

The title compound, C_34_H_28_N_6_O_2_, has an inversion centre located at the mid-point of the central C—C bond of the diether bridging unit. The central pyridine rings of the terpyridyl units and the diether chain are co-planar: the maximum deviation from the 18-atom mean plane defined by the bridging unit and the central pyridyl ring is for the pyridyl N atom which sits 0.055 (1) Å above the plane. The dihedral angles between the terminal pyridine rings with this plane are 10.3 (1) and 37.6 (1)°, repectively. In the crystal, weak C—H⋯N inter­actions link the mol­ecules into infinite chains parallel to the *a* axis.

## Related literature

For the structure of the unsubstituted 2,2′:6′,2′′-terpyridine compound, see: Bessel *et al.* (1992 ▶). For the structure of the precursor of the title compound, 4′-chloro-2,2′: 6′,2′′-terpyridine, see: Beves *et al.* (2006 ▶). For reviews of functionalized 2,2′:6′,2′′-terpyridine compounds, see: Fallahpour (2003 ▶); Heller & Schubert (2003 ▶). For a comprehensive review of platinum terpyridines, see: Newkome *et al.* (2008 ▶). For the structure of bis­(2,2′:6′,2′′-terpyrid-4′-yl) ether, see: Constable *et al.* (1995 ▶). For the synthesis of the title compound, see: Van der Schilden (2006 ▶); Constable *et al.* (2005 ▶). For the synthesis and structures of related bis­(terpy) structures linked by an alk­oxy substituent, see: Constable *et al.* (2006 ▶).
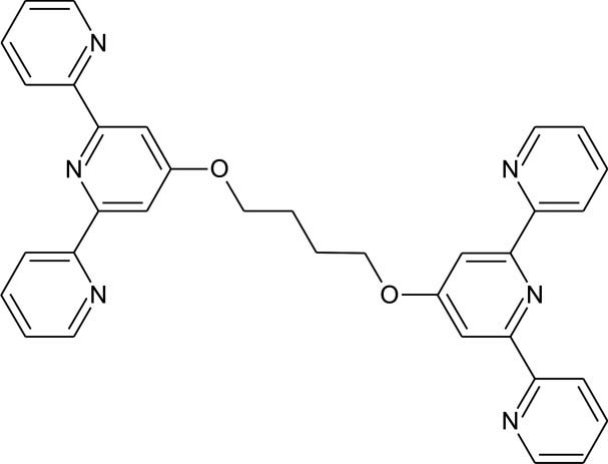


## Experimental

### 

#### Crystal data

C_34_H_28_N_6_O_2_
*M* *_r_* = 552.62
Triclinic, 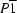

*a* = 6.3678 (2) Å
*b* = 10.5088 (4) Å
*c* = 10.9216 (3) Å
α = 72.580 (3)°
β = 78.561 (3)°
γ = 77.438 (3)°
*V* = 673.64 (4) Å^3^
*Z* = 1
Mo *K*α radiation
μ = 0.09 mm^−1^
*T* = 100 K
0.40 × 0.40 × 0.30 mm

#### Data collection

Oxford Diffraction Xcalibur 2 CCD diffractometer
Absorption correction: multi-scan (Blessing, 1995 ▶) *T*_min_ = 0.966, *T*_max_ = 0.974
10771 measured reflections
4604 independent reflections
3678 reflections with *I* > 2σ(*I*)
*R*_int_ = 0.023

#### Refinement

*R*[*F* ^2^ > 2σ(*F* ^2^)] = 0.053
*wR*(*F* ^2^) = 0.148
*S* = 1.04
4604 reflections
190 parameters
H-atom parameters constrained
Δρ_max_ = 0.46 e Å^−3^
Δρ_min_ = −0.43 e Å^−3^

### 

Data collection: *CrysAlis CCD* (Oxford Diffraction, 2008 ▶); cell refinement: *CrysAlis CCD*; data reduction: *CrysAlis RED* (Oxford Diffraction, 2008 ▶); program(s) used to solve structure: *SHELXS97* (Sheldrick, 2008 ▶); program(s) used to refine structure: *SHELXL97* (Sheldrick, 2008 ▶); molecular graphics: *ORTEP-3* (Farrugia, 1997 ▶); software used to prepare material for publication: *WinGX* (Farrugia, 1999 ▶).

## Supplementary Material

Crystal structure: contains datablock(s) I, global. DOI: 10.1107/S1600536811050343/lr2036sup1.cif

Structure factors: contains datablock(s) I. DOI: 10.1107/S1600536811050343/lr2036Isup2.hkl

Supplementary material file. DOI: 10.1107/S1600536811050343/lr2036Isup3.cml

Additional supplementary materials:  crystallographic information; 3D view; checkCIF report

## Figures and Tables

**Table 1:** Hydrogen-bond geometry (Å, °)

*D*—H⋯*A*	*D*—H	H⋯*A*	*D*⋯*A*	*D*—H⋯*A*
C13—H13⋯N3^i^	0.95	2.66	3.592 (1)	168

Symmetry code: (i) 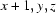.

**Fig. 1.**
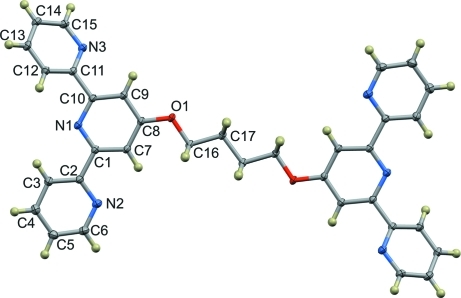
The molecular structure of the title compound. Displacement ellipsoids are drawn at the 40% probability level.

**Fig. 2.**
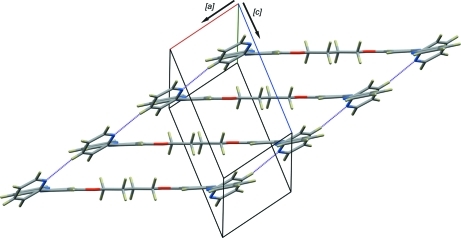
A view of packing of the title compound.

## supplementary crystallographic information

### Comment

The title compound is one in a series of ligands developed in an attempt to harness multifunctional activity. Upon coordination to platinum(II) these complexes should be able to covalently bind DNA through both metal centres, thus increasing the number of adducts formed. Furthermore the presence of the flexible diol derived linkage will provide the complex with the potential to engage in long range interactions with DNA.

The ligand crystallized in the triclinic space group P-1, with a half molecule in the asymmetric unit and *Z* = 1. The two halves of the ligand are related by crystallographically imposed inversion symmetry. The inversion centre is located at the mid-point of the di-ether linkage unit. The three pyridine rings adopt a *trans*, *trans* conformation as observed in the parent 4'-chloro-2,2':6',2''- terpyridine (Beves *et al.*, 2006) and in uncoordinated terpy ligands in general.

The central pyridine rings of the terpy ligands are in the same plane as the bridging di-ether chain. The terminal pyridine rings of the terpyridine ligand are, however canted relative to the central pyridine ring. The C9–C10–C11–N3 torsion angle is 35.4 (1)°, while the C7–C2–C1–N2 torsion angle is 7.1 (1)° (refer to Figure 1 for the atom numbering scheme). The large torsion angle of the pyridine ring containing N3 is seemingly to allow for hydrogen bonding between the pyridine nitrogen atom N3 and the H13 hydrogen atom of an adjacent molecule (Table 1) . This interaction links the molecules into a one-dimensional chain (Figure 2) along the *a*-axis direction. There is no indication of meaningful π···π or C–H···π interactions in the crystal, which are often observed in terpyridine structures (Beves *et al.*, 2006).

### Experimental

The title compound was prepared by an adaptation of a previously described method (Van der Schilden, 2006 and Constable *et al.*, 2005). Butanediol (1.13 mmol) was added to a suspension of ground potassium hydroxide (6.69 mmol) in DMSO (30 ml). The solution was heated to reflux for 1 h after which 4'-chloro-2,2':6',2''-terpyridine (2.23 mmol) was added. The mixture was again brought to reflux for an additional 24 h. After cooling to room temperature, the brown mixture was added to cold water (100 ml). The resulting off-white precipitate was filtered, rinsed with cold ethanol and air dried. Single crystals of were grown by slow liquid diffusion of n-hexane into a chloroform solution of the compound.

### Refinement

All non-hydrogen atoms were located in the difference Fourier map and refined anisotropically. The positions of all hydrogen atoms were calculated using the standard riding model of *SHELXL97.* with C—H(aromatic)and C—H (methylene)distances of 0.93 Å and *U*_iso_ = 1.2 *U*_eq_.

### Crystal data

**Table:**

C_34_H_28_N_6_O_2_	*Z* = 1
*M**_r_* = 552.62	*F*(000) = 290
Triclinic, *P*1	*D*_x_ = 1.362 Mg m^−^^3^
Hall symbol: -P 1	Mo *K*α radiation, λ = 0.71073 Å
*a* = 6.3678 (2) Å	Cell parameters from 4604 reflections
*b* = 10.5088 (4) Å	θ = 3.2–34.2°
*c* = 10.9216 (3) Å	µ = 0.09 mm^−^^1^
α = 72.580 (3)°	*T* = 100 K
β = 78.561 (3)°	Triangular, colourless
γ = 77.438 (3)°	0.40 × 0.40 × 0.30 mm
*V* = 673.64 (4) Å^3^	

### Data collection

**Table:**

Oxford Diffraction Xcalibur 2 CCD diffractometer	4604 independent reflections
Radiation source: fine-focus sealed tube	3678 reflections with *I* > 2σ(*I*)
graphite	*R*_int_ = 0.023
ω scans at fixed θ angles	θ_max_ = 34.2°, θ_min_ = 3.2°
Absorption correction: multi-scan (Blessing, 1995)	*h* = −10→9
*T*_min_ = 0.966, *T*_max_ = 0.974	*k* = −15→15
10771 measured reflections	*l* = −15→16

### Refinement

**Table:**

Refinement on *F*^2^	Primary atom site location: structure-invariant direct methods
Least-squares matrix: full	Secondary atom site location: difference Fourier map
*R*[*F*^2^ > 2σ(*F*^2^)] = 0.053	Hydrogen site location: inferred from neighbouring sites
*wR*(*F*^2^) = 0.148	H-atom parameters constrained
*S* = 1.04	*w* = 1/[σ^2^(*F*_o_^2^) + (0.1033*P*)^2^] where *P* = (*F*_o_^2^ + 2*F*_c_^2^)/3
4604 reflections	(Δ/σ)_max_ = 0.001
190 parameters	Δρ_max_ = 0.46 e Å^−^^3^
0 restraints	Δρ_min_ = −0.43 e Å^−^^3^

### Special details

**Table:**

Geometry. All s.u.'s (except the s.u. in the dihedral angle between two l.s. planes) are estimated using the full covariance matrix. The cell s.u.'s are taken into account individually in the estimation of s.u.'s in distances, angles and torsion angles; correlations between s.u.'s in cell parameters are only used when they are defined by crystal symmetry. An approximate (isotropic) treatment of cell s.u.'s is used for estimating s.u.'s involving l.s. planes.
Refinement. Refinement of *F*^2^ against ALL reflections. The weighted *R*-factor *wR* and goodness of fit *S* are based on *F*^2^, conventional *R*-factors *R* are based on *F*, with *F* set to zero for negative *F*^2^. The threshold expression of *F*^2^ > 2σ(*F*^2^) is used only for calculating *R*-factors(gt) *etc*. and is not relevant to the choice of reflections for refinement. *R*-factors based on *F*^2^ are statistically about twice as large as those based on *F*, and *R*- factors based on ALL data will be even larger.

### Fractional atomic coordinates and isotropic or equivalent isotropic displacement parameters (Å^2^)

**Table:**

	*x*	*y*	*z*	*U*_iso_*/*U*_eq_	
C1	0.91969 (13)	0.45081 (8)	0.20750 (8)	0.01459 (17)	
C2	0.99907 (14)	0.57692 (9)	0.12469 (8)	0.01538 (17)	
C3	1.21800 (15)	0.57653 (9)	0.07304 (9)	0.01893 (18)	
H3	1.3198	0.4946	0.0869	0.023*	
C4	1.28436 (16)	0.69785 (10)	0.00112 (9)	0.0218 (2)	
H4	1.4330	0.7009	−0.0331	0.026*	
C5	1.12985 (16)	0.81461 (10)	−0.01999 (9)	0.0227 (2)	
H5	1.1698	0.8990	−0.0699	0.027*	
C6	0.91517 (17)	0.80504 (9)	0.03359 (10)	0.0231 (2)	
H6	0.8093	0.8850	0.0179	0.028*	
C7	0.70581 (14)	0.45958 (9)	0.27116 (8)	0.01544 (17)	
H7	0.6095	0.5438	0.2593	0.019*	
C8	0.63836 (13)	0.34181 (9)	0.35221 (8)	0.01472 (17)	
C9	0.78076 (13)	0.21929 (8)	0.36168 (8)	0.01555 (17)	
H9	0.7374	0.1368	0.4146	0.019*	
C10	0.98796 (13)	0.22108 (8)	0.29156 (8)	0.01479 (17)	
C11	1.14276 (14)	0.09254 (8)	0.29357 (8)	0.01510 (17)	
C12	1.36485 (14)	0.08795 (9)	0.28696 (9)	0.01945 (19)	
H12	1.4194	0.1663	0.2839	0.023*	
C13	1.50515 (15)	−0.03274 (10)	0.28492 (10)	0.0221 (2)	
H13	1.6569	−0.0394	0.2832	0.026*	
C14	1.41963 (15)	−0.14337 (9)	0.28550 (10)	0.0225 (2)	
H14	1.5118	−0.2265	0.2808	0.027*	
C15	1.19601 (15)	−0.12984 (9)	0.29316 (10)	0.0213 (2)	
H15	1.1382	−0.2063	0.2943	0.026*	
C16	0.29097 (13)	0.46356 (9)	0.41509 (9)	0.01583 (17)	
H16A	0.2626	0.5046	0.3244	0.019*	
H16B	0.3543	0.5275	0.4417	0.019*	
C17	0.08210 (13)	0.43460 (8)	0.50317 (8)	0.01595 (18)	
H17A	0.1122	0.3928	0.5934	0.019*	
H17B	0.0207	0.3699	0.4764	0.019*	
N1	1.06151 (11)	0.33489 (7)	0.21789 (7)	0.01511 (16)	
N2	0.84919 (13)	0.68991 (8)	0.10593 (8)	0.02008 (17)	
N3	1.05674 (12)	−0.01556 (7)	0.29902 (8)	0.01867 (17)	
O1	0.43840 (10)	0.33755 (6)	0.42503 (6)	0.01732 (15)	

### Atomic displacement parameters (Å^2^)

**Table:**

	*U*^11^	*U*^22^	*U*^33^	*U*^12^	*U*^13^	*U*^23^
C1	0.0132 (4)	0.0138 (4)	0.0159 (4)	0.0007 (3)	−0.0025 (3)	−0.0045 (3)
C2	0.0153 (4)	0.0139 (4)	0.0154 (4)	0.0003 (3)	−0.0020 (3)	−0.0036 (3)
C3	0.0158 (4)	0.0180 (4)	0.0202 (4)	−0.0006 (3)	−0.0003 (3)	−0.0040 (3)
C4	0.0197 (4)	0.0214 (4)	0.0222 (4)	−0.0047 (3)	0.0010 (3)	−0.0045 (4)
C5	0.0271 (5)	0.0171 (4)	0.0220 (5)	−0.0058 (4)	−0.0014 (4)	−0.0022 (4)
C6	0.0243 (5)	0.0146 (4)	0.0262 (5)	0.0005 (3)	−0.0026 (4)	−0.0026 (4)
C7	0.0129 (4)	0.0137 (4)	0.0182 (4)	0.0020 (3)	−0.0021 (3)	−0.0051 (3)
C8	0.0107 (3)	0.0156 (4)	0.0171 (4)	0.0004 (3)	−0.0016 (3)	−0.0053 (3)
C9	0.0122 (4)	0.0135 (4)	0.0190 (4)	0.0004 (3)	−0.0019 (3)	−0.0034 (3)
C10	0.0118 (3)	0.0137 (4)	0.0182 (4)	0.0012 (3)	−0.0033 (3)	−0.0050 (3)
C11	0.0125 (4)	0.0133 (4)	0.0172 (4)	0.0007 (3)	−0.0016 (3)	−0.0031 (3)
C12	0.0130 (4)	0.0156 (4)	0.0282 (5)	0.0004 (3)	−0.0035 (3)	−0.0053 (4)
C13	0.0128 (4)	0.0187 (4)	0.0298 (5)	0.0024 (3)	−0.0019 (3)	−0.0035 (4)
C14	0.0181 (4)	0.0149 (4)	0.0289 (5)	0.0040 (3)	−0.0016 (4)	−0.0038 (4)
C15	0.0194 (4)	0.0131 (4)	0.0285 (5)	0.0003 (3)	−0.0028 (4)	−0.0039 (4)
C16	0.0115 (4)	0.0151 (4)	0.0188 (4)	0.0020 (3)	−0.0022 (3)	−0.0043 (3)
C17	0.0119 (4)	0.0161 (4)	0.0184 (4)	0.0003 (3)	−0.0016 (3)	−0.0049 (3)
N1	0.0127 (3)	0.0130 (3)	0.0181 (3)	0.0010 (2)	−0.0024 (3)	−0.0041 (3)
N2	0.0186 (4)	0.0143 (3)	0.0238 (4)	0.0015 (3)	−0.0022 (3)	−0.0033 (3)
N3	0.0149 (3)	0.0135 (3)	0.0252 (4)	−0.0001 (3)	−0.0024 (3)	−0.0034 (3)
O1	0.0111 (3)	0.0143 (3)	0.0224 (3)	0.0022 (2)	0.0010 (2)	−0.0039 (3)

### Geometric parameters (Å, °)

**Table:**

C1—N1	1.3385 (10)	C10—N1	1.3473 (11)
C1—C7	1.3982 (12)	C10—C11	1.4860 (12)
C1—C2	1.4907 (12)	C11—N3	1.3459 (11)
C2—N2	1.3423 (11)	C11—C12	1.3927 (12)
C2—C3	1.3960 (12)	C12—C13	1.3869 (12)
C3—C4	1.3871 (13)	C12—H12	0.9500
C3—H3	0.9500	C13—C14	1.3848 (13)
C4—C5	1.3865 (13)	C13—H13	0.9500
C4—H4	0.9500	C14—C15	1.3881 (13)
C5—C6	1.3887 (14)	C14—H14	0.9500
C5—H5	0.9500	C15—N3	1.3390 (11)
C6—N2	1.3348 (12)	C15—H15	0.9500
C6—H6	0.9500	C16—O1	1.4354 (10)
C7—C8	1.3872 (12)	C16—C17	1.5101 (12)
C7—H7	0.9500	C16—H16A	0.9900
C8—O1	1.3629 (10)	C16—H16B	0.9900
C8—C9	1.3930 (11)	C17—C17^i^	1.5278 (16)
C9—C10	1.3889 (12)	C17—H17A	0.9900
C9—H9	0.9500	C17—H17B	0.9900
			
N1—C1—C7	123.79 (8)	N3—C11—C10	116.45 (7)
N1—C1—C2	117.13 (7)	C12—C11—C10	120.57 (8)
C7—C1—C2	119.07 (8)	C13—C12—C11	118.97 (8)
N2—C2—C3	122.59 (8)	C13—C12—H12	120.5
N2—C2—C1	116.08 (8)	C11—C12—H12	120.5
C3—C2—C1	121.31 (8)	C14—C13—C12	118.66 (8)
C4—C3—C2	118.85 (9)	C14—C13—H13	120.7
C4—C3—H3	120.6	C12—C13—H13	120.7
C2—C3—H3	120.6	C13—C14—C15	118.40 (8)
C5—C4—C3	118.82 (9)	C13—C14—H14	120.8
C5—C4—H4	120.6	C15—C14—H14	120.8
C3—C4—H4	120.6	N3—C15—C14	124.01 (8)
C4—C5—C6	118.33 (8)	N3—C15—H15	118.0
C4—C5—H5	120.8	C14—C15—H15	118.0
C6—C5—H5	120.8	O1—C16—C17	107.75 (7)
N2—C6—C5	123.71 (9)	O1—C16—H16A	110.2
N2—C6—H6	118.1	C17—C16—H16A	110.2
C5—C6—H6	118.1	O1—C16—H16B	110.2
C8—C7—C1	118.07 (8)	C17—C16—H16B	110.2
C8—C7—H7	121.0	H16A—C16—H16B	108.5
C1—C7—H7	121.0	C16—C17—C17^i^	110.24 (9)
O1—C8—C7	123.93 (8)	C16—C17—H17A	109.6
O1—C8—C9	116.87 (7)	C17^i^—C17—H17A	109.6
C7—C8—C9	119.20 (8)	C16—C17—H17B	109.6
C10—C9—C8	118.14 (8)	C17^i^—C17—H17B	109.6
C10—C9—H9	120.9	H17A—C17—H17B	108.1
C8—C9—H9	120.9	C1—N1—C10	116.83 (7)
N1—C10—C9	123.81 (8)	C6—N2—C2	117.68 (8)
N1—C10—C11	115.91 (7)	C15—N3—C11	116.94 (8)
C9—C10—C11	120.27 (8)	C8—O1—C16	117.00 (7)
N3—C11—C12	122.95 (8)		
			
N1—C1—C2—N2	174.04 (7)	C9—C10—C11—C12	−146.06 (9)
C7—C1—C2—N2	−7.06 (12)	N3—C11—C12—C13	0.47 (15)
N1—C1—C2—C3	−7.51 (12)	C10—C11—C12—C13	−177.97 (8)
C7—C1—C2—C3	171.39 (8)	C11—C12—C13—C14	1.97 (15)
N2—C2—C3—C4	1.01 (14)	C12—C13—C14—C15	−2.45 (15)
C1—C2—C3—C4	−177.34 (8)	C13—C14—C15—N3	0.56 (16)
C2—C3—C4—C5	−1.79 (14)	O1—C16—C17—C17^i^	179.99 (8)
C3—C4—C5—C6	0.89 (14)	C7—C1—N1—C10	1.97 (12)
C4—C5—C6—N2	0.94 (15)	C2—C1—N1—C10	−179.19 (7)
N1—C1—C7—C8	1.61 (13)	C9—C10—N1—C1	−3.86 (12)
C2—C1—C7—C8	−177.21 (7)	C11—C10—N1—C1	175.46 (7)
C1—C7—C8—O1	176.41 (7)	C5—C6—N2—C2	−1.74 (15)
C1—C7—C8—C9	−3.43 (12)	C3—C2—N2—C6	0.74 (13)
O1—C8—C9—C10	−178.11 (7)	C1—C2—N2—C6	179.17 (8)
C7—C8—C9—C10	1.73 (12)	C14—C15—N3—C11	1.81 (15)
C8—C9—C10—N1	2.05 (13)	C12—C11—N3—C15	−2.32 (14)
C8—C9—C10—C11	−177.24 (7)	C10—C11—N3—C15	176.17 (8)
N1—C10—C11—N3	−143.94 (8)	C7—C8—O1—C16	0.40 (12)
C9—C10—C11—N3	35.40 (11)	C9—C8—O1—C16	−179.76 (7)
N1—C10—C11—C12	34.59 (12)	C17—C16—O1—C8	179.98 (7)

Symmetry codes: (i) −*x*, −*y*+1, −*z*+1.

### Hydrogen-bond geometry (Å, °)

**Table:**

*D*—H···*A*	*D*—H	H···*A*	*D*···*A*	*D*—H···*A*
C13—H13···N3^ii^	0.95	2.66	3.592 (1)	168.

Symmetry codes: (ii) *x*+1, *y*, *z*.
